# Re-evaluation of different electrophysiological criteria for Guillain-Barré syndrome in a single cohort from China

**DOI:** 10.3389/fneur.2026.1766901

**Published:** 2026-03-11

**Authors:** Qiongqiong Zhai, Cheng Guo, Zihan Gao, Fang Xue

**Affiliations:** 1 Department of Neurology, The Second Hospital of Hebei Medical University, Shijiazhuang, China; 2 Department of Pediatrics, The Third Hospital of Hebei Medical University, Shijiazhuang, China; 3 Department of Cardiovascular, The Second Hospital of Hebei Medical University, Shijiazhuang, China

**Keywords:** acute inflammatory demyelinating polyneuropathy, acute motor axonal neuropathy, conduction block, electrophysiological criteria, Guillain-Barré syndrome

## Abstract

**Background and aims:**

We aimed to compare electrophysiological subtypes of Guillain-Barré syndrome (GBS) in a single cohort from northern China using three criteria (Ho’s, Hadden’s, Rajabally’s), to clarify whether GBS subtypes are affected by electrophysiological criteria and nerve conduction studies (NCS) examination timing, to explore the value of sural sparing in subtype classification, and analyze the association between conduction block (CB) and short-term prognosis of Acute Motor Axonal Neuropathy (AMAN).

**Methods:**

We retrospectively collected GBS patients hospitalized in the Department of Neurology, the Second Hospital of Hebei Medical University (Jan 2017–Jan 2022), who met Brighton diagnostic criteria and had complete NCS data. Patients were classified via three electrophysiological criteria (Ho’s, Hadden’s, Rajabally’s); we analyzed subtype distribution by NCS timing (1, 2, or ≥3 weeks post-onset) and used discharge Hughes Functional Grading Scale (HFGS) to assess short-term prognosis.

**Results:**

A total of 262 patients were enrolled. AMAN was the main subtype (40.1% by Hadden’s, 46.6% by Ho’s, 59.5% by Rajabally’s criteria). Fleiss Kappa showed strong consistency among the three electrophysiological criteria (*κ* = 0.75, *p* < 0.001); Subtype composition showed greater consistency at ≥3 weeks post-onset (*p* > 0.05). Acute Inflammatory Demyelinating Polyneuropathy (AIDP)had higher sural sparing than AMAN (32.4%–50% vs. 0.9%–4%, *p* < 0.001). AMAN with CB had lower discharge HFGS (*p* = 0.012). Serial NCS enhanced the accuracy of GBS subtype classification.

**Conclusion:**

GBS electrodiagnosis depends on criteria and NCS timing (≥3 weeks more consistent). AMAN is predominant in our cohort from northern China regardless of criteria used. Sural sparing and serial NCS enhance subtype classification accuracy.

## 1 Introduction

Guillain-Barré syndrome (GBS) is an immune-mediated acute inflammatory peripheral neuropathy (1). Acute Inflammatory Demyelinating Polyneuropathies (AIDP) and Acute Motor Axonal Neuropathy (AMAN) are the two main subtypes of GBS, and the electrophysiological classification of GBS mainly relies on electrophysiological examination (2, 3). Accurately distinguishing subtypes of GBS may improve their clinical prognosis.

Multiple GBS electrophysiological criteria (2–6) have been proposed for GBS classification. There is still controversy over whether the electrophysiological subtypes of GBS can be diagnosed based on a single electrophysiological examination, and which standard sets and critical values should be used (7, 8). It has been found that AMAN patients may experience transient conduction block or conduction slowing, simulating demyelination electrophysiological manifestations but without abnormal temporal dispersion (9–11) (i.e., Reversible conduction failure, RCF). Based on the traditional diagnostic criteria [Ho’s criteria (2) and Hadden’s criteria (3)], 24%–38% of patients with AMAN may be incorrectly classified as AIDP (12–14). The Rajabally’s criteria (4) encompassed RCF within the diagnostic criteria of AMAN, enhanced the diagnostic requirements for AIDP. Unicin et al. (15) found that the Rajabally’s criteria enhanced the sensitivity of AMAN but reduced the sensitivity of AIDP. It was reported that serial electrophysiological examinations serve as a key gold standard for the subtype classification of GBS (16, 17). In addition, all current classification criteria do not involve sensory nerve conduction tests. However, some studies have suggested that sensory nerve conduction, such as the sural sparing, may aid in GBS subtype classification and should be considered in future classification criteria (18).

At present, there is a lack of comparative studies on different electrophysiological classification standards. AMAN was first reported in northern China and was the main subtype of GBS in northern China, while AIDP was the main subtype of GBS in other regions of China. However, it remains unclear whether different classification criteria and electrophysiological examination timing affect GBS subtyping, whether sensory nerve conduction (e.g., sural sparing) aids in classification and whether RCF has a predictive effect on the clinical prognosis of GBS. Therefore, we retrospectively studied the clinical and electrophysiological data of patients with GBS, employing various electrophysiological classification criteria for comparative research. This is the first study to quantitatively evaluate these electrophysiological criteria for GBS in a single cohort from northern China.

## 2 Materials and methods

### 2.1 Patients

We retrospectively collected clinical data about GBS patients hospitalized in the Department of Neurology and Pediatrics of the Second Hospital of Hebei Medical University from January 1, 2017 to January 1, 2022, who met the inclusion and exclusion criteria.

Inclusion criteria: (1) Patients diagnosed with GBS for the first time. (2) Brighton diagnostic criteria (19) were used to diagnose GBS: The natural course of the disease was consistent with GBS and other diagnoses for limb weakness were ruled out. The core clinical features include a monophasic course with an interval of 12 h to 28 days between onset and peak clinical symptoms, bilateral and flaccid limb weakness, weakened or absent tendon reflexes, cerebrospinal fluid albumin cell separation, and nerve conduction test results as auxiliary examinations to support diagnosis. (3) For those who met the core clinical manifestations of GBS but retained tendon reflexes upon admission and those whose auxiliary examinations were consistent with GBS characteristics, excluding other diagnoses, they were also included.

Exclusion criteria: Patients who had chronic inflammatory demyelinating polyneuropathy (CIDP) (with a course of disease > 8 weeks, progressive demyelinating changes or chronic demyelinating markers), spinal cord disease, peripheral neuropathy caused by other causes (diabetes, vasculitis, toxicity, drug) were excluded.

We collected general clinical data, laboratory test results, and nerve conduction study (NCS) results.

### 2.2 Electrophysiological examination

① We used a Dantec Keypoint electromyography/evoked potential device for electrophysiological examinations. The NCS of all patients included at least four motor nerves (median nerve, ulnar nerve, common peroneal nerve, and tibial nerve) and three sensory nerves (median nerve, ulnar nerve, and sural nerve). At least two F-waves were detected in the upper and lower limbs and needle electromyography was performed in patients who could cooperate. Nerve conduction tests were completed by a specialist in neurophysiology in our hospital’s electromyography department.

② Conduction Block (CB): CB was diagnosed in accordance with the 2020 guidelines of the American Association of Electrodiagnostic Medicine (AANEM) (20), with the following specific criteria: ① For standard motor nerve segments (e.g., median nerve elbow-wrist, ulnar nerve elbow-wrist, common peroneal nerve knee-ankle), the compound muscle action potential (CMAP) amplitude at the proximal stimulation site was reduced by ≥50% compared with the distal site, while the waveform duration increased by ≤30% (to exclude temporal dispersion as the main cause of amplitude reduction); ② Before confirming CB, technical factors (e.g., insufficient stimulation intensity, electrode displacement, skin resistance >5 kΩ) and physiological factors (e.g., nerve compression at the stimulation site) were strictly excluded.

③ Sural sparing (SS): There are at least two abnormal upper limb sensory nerve action potentials (SNAPs) (median, ulnar, and radial nerve SNAPs) present, while lower limb sural nerve SNAPs are normal or relatively preserved (21). For GBS patients, median/ulnar nerve conduction tests are routine exams while radial nerve tests are not. As a retrospective study which was limited by the availability of routine tests (incomplete radial nerve data), the inclusion criteria for sural sparing were set as: two abnormal upper limb SNAPs (involving the median/ulnar nerves) + normal or relatively preserved lower limb sural SNAPs.

### 2.3 Comparison of three electrophysiological criteria

Two neurologists (QZ, FX) concealed the clinical information of patients and classified the NCS results of GBS patients based on Ho criteria (2), Hadden criteria (3), and Rajabally criteria (4). Key differences between the three criteria are as follows: Ho’s criteria use “exact time dispersion” (without clear definition) to diagnose AIDP. Hadden’s criteria replace “waveform dispersion” with “CB” for AIDP diagnosis. Rajabally’s criteria impose stricter requirements for AIDP and allow reversible CB in AMAN (see Supplementary Table 1 for full details). The subtype classification of GBS patients includes AIDP, AMAN, Inexcitable, Equivocal, and Normal.

### 2.4 GBS serial electrophysiological examination

For serial electrophysiological analysis, we included GBS patients who underwent two NCS tests: the first within 2 weeks post-onset and the second between 3 and 8 weeks post-onset. A total of 31 patients met this criterion and were analyzed for subtype changes.

### 2.5 Ethical approval

This study had been approved by the Research Ethics Committee of the Second Hospital of Hebei Medical University (Ethics Batch Number: 2022-R572). Due to the retrospective nature of the study, informed consent was waived.

### 2.6 Statistical analyses

Statistical analysis and plotting were performed using SPSS version 22.0 (IBM, New York, USA) and Microsoft Power BI software (Microsoft, Washington State, USA). Count data was expressed as the number of cases or composition ratio *n* (%) and comparisons of composition ratios were performed using the chi-square test or Fisher’s exact test. Fleiss Kappa test (via the Fleiss Kappa plugin in SPSS) was used to evaluate the consistency of each electrophysiological criterion. A two-tailed *p*-value < 0.05 indicated a statistically significant difference. Microsoft Power BI software was used to draw Sankey diagrams illustrating patients’ transition patterns among different electrophysiological criteria.

## 3 Results

### 3.1 General information

**Figure 1:**
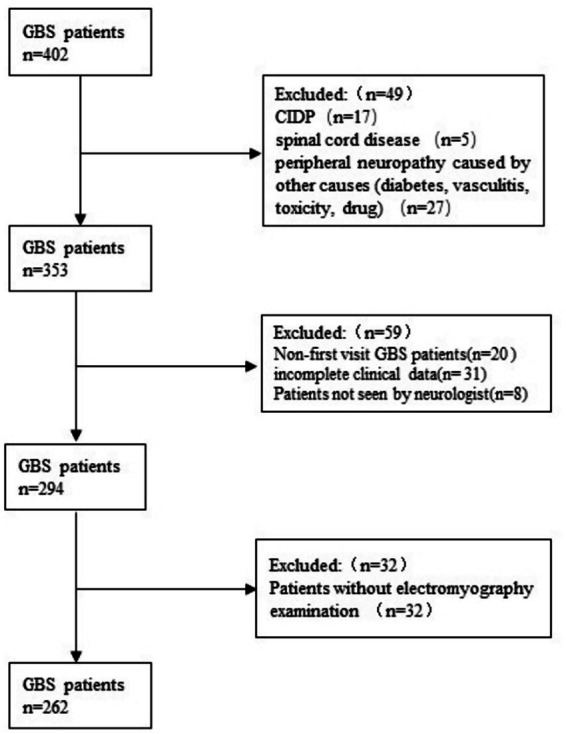
Patients flow diagram.

We collected 294 patients with GBS and the clinical data of the 294 patients were detailed in our previous study (22). Among the 294 GBS patients, 262 patients underwent NCS (see Figure 1). The average time from the onset of clinical symptoms to the first NCS assessment was 11.84 days (range 2–31 days). This retrospective study showed that no GBS patients experienced disease progression during 8 weeks of outpatient and inpatient follow-up, and their clinical features did not meet the diagnostic criteria for CIDP and other causes of peripheral neuropathy.

### 3.2 Comparison of GBS subtypes based on different electrophysiological criteria

Electrophysiological typing was performed on 262 GBS patients using the Hadden criteria, Ho criteria, and Rajabally criteria (see Table 1). The results showed that AMAN subtype was the main subtype of GBS patients (40.1%, 46.6%, and 59.5%, respectively) in our cohort. According to the Rajabally criteria, the proportion of AMAN was the highest (59.5%). Among the three criteria, the proportion of AIDP obtained based on the Hadden criteria was the highest (37.8%). The Fleiss Kappa test showed strong consistency among the three electrophysiological criteria results (kappa value 0.75, *p* < 0.0001).

**Table 1:** Comparison of 262 GBS patients with different electrophysiological criteria.

GBS subtypes	Hadden’s criteria	Ho’s criteria	Rajabally’s criteria	Kappa test	*p-value*
AIDP, *n*%	99 (37.8)	77 (29.4)	50 (19.1)	0.683	<0.0001
AMAN, *n* (%)	105 (40.1)	122 (46.6)	156 (59.5)	0.730	<0.0001
Equivocal, *n* (%)	28 (10.7)	33 (12.6)	26 (9.9)	0.677	<0.0001
Inexcitable, *n* (%)	6 (2.3)	6 (2.3)	6 (2.3)	1.000	<0.0001
Normal, *n* (%)	24 (9.1)	24 (9.1)	24 (9.2)	1.000	<0.0001

AIDP, acute inflammatory demyelinating polyneuropathy; AMAN, acute motor axonal neuropathy.

### 3.3 Changes in different electrophysiological subtypes

**Figure 2:**
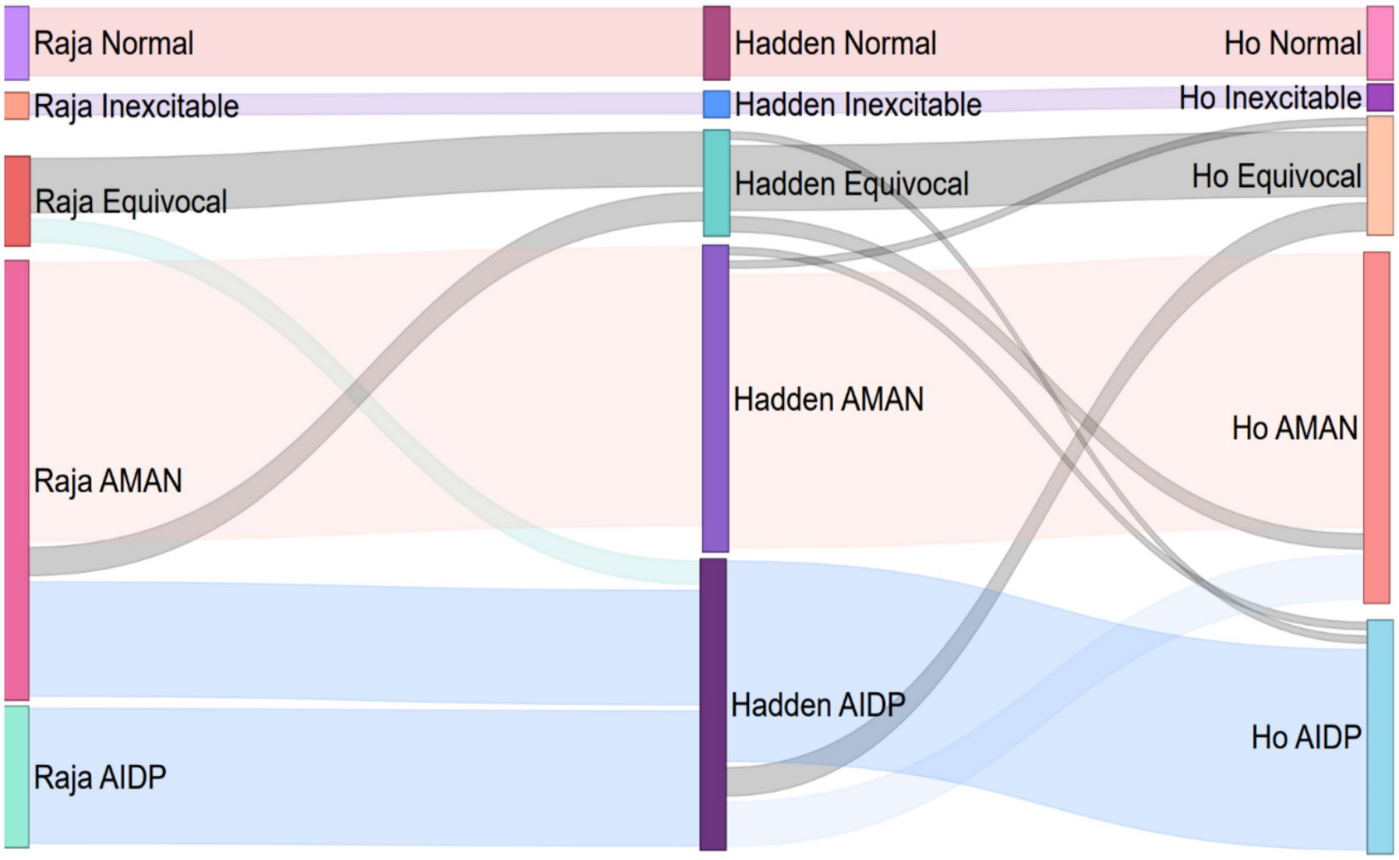
Transformation of the classification base on different electrodiagnostic criteria. Raja, Rajabally’s criteria; Hadden, Hadden’s criteria; Ho, Ho’s criteria; Different classification criteria lead to shifts in electrophysiological subtypes among patients with GBS.

Different classification criteria lead to shifts in electrophysiological subtypes among patients with GBS (see Figure 2). Per Rajabally’s criteria, some Hadden-classified AIDP patients were reclassified as AMAN (*n* = 42) or Equivocal (*n* = 7), and some Hadden-classified Equivocal patients as AMAN (*n* = 9). Per Ho’s criteria, some Hadden-classified AIDP patients became AMAN (*n* = 15) or Equivocal (*n* = 9). A few Hadden-classified AMAN patients shifted to AIDP (*n* = 1) or Equivocal (*n* = 1) and some Hadden-classified Equivocal patients to AIDP (*n* = 1) or AMAN (*n* = 4) with Ho’s criteria.

### 3.4 The influence of NCS timing on electrophysiological subtypes

**Figure 3:**
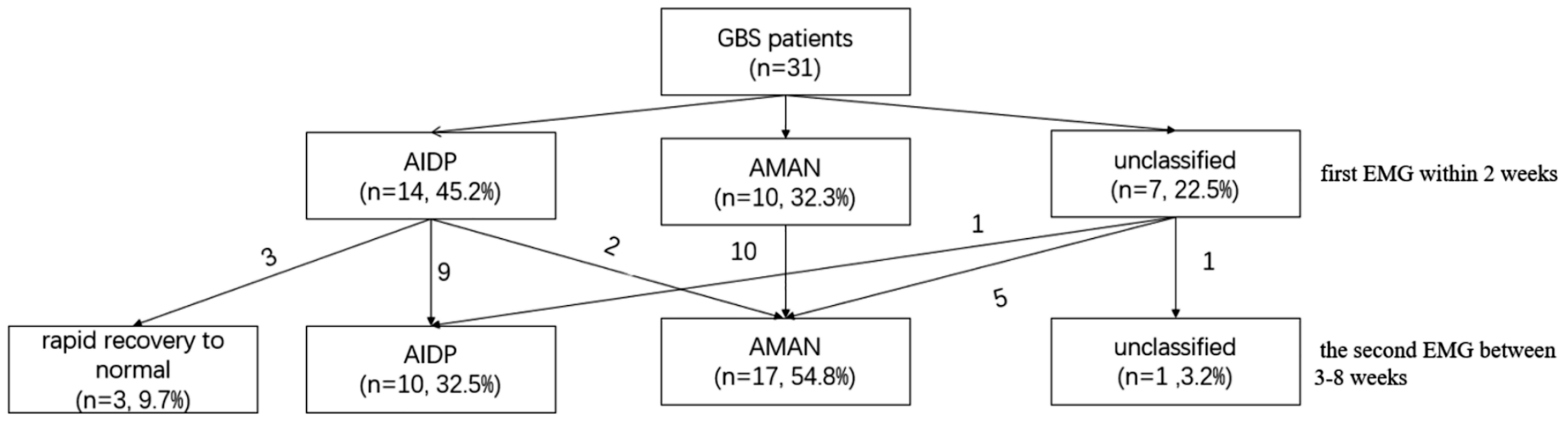
Classification change of GBS patients after serial NCS. Thirty-one GBS patients had ≥2 serial follow-up electrophysiological tests, where the first test was within 2 weeks and the second between 3 and 8 weeks after onset.

**Figure 4:**
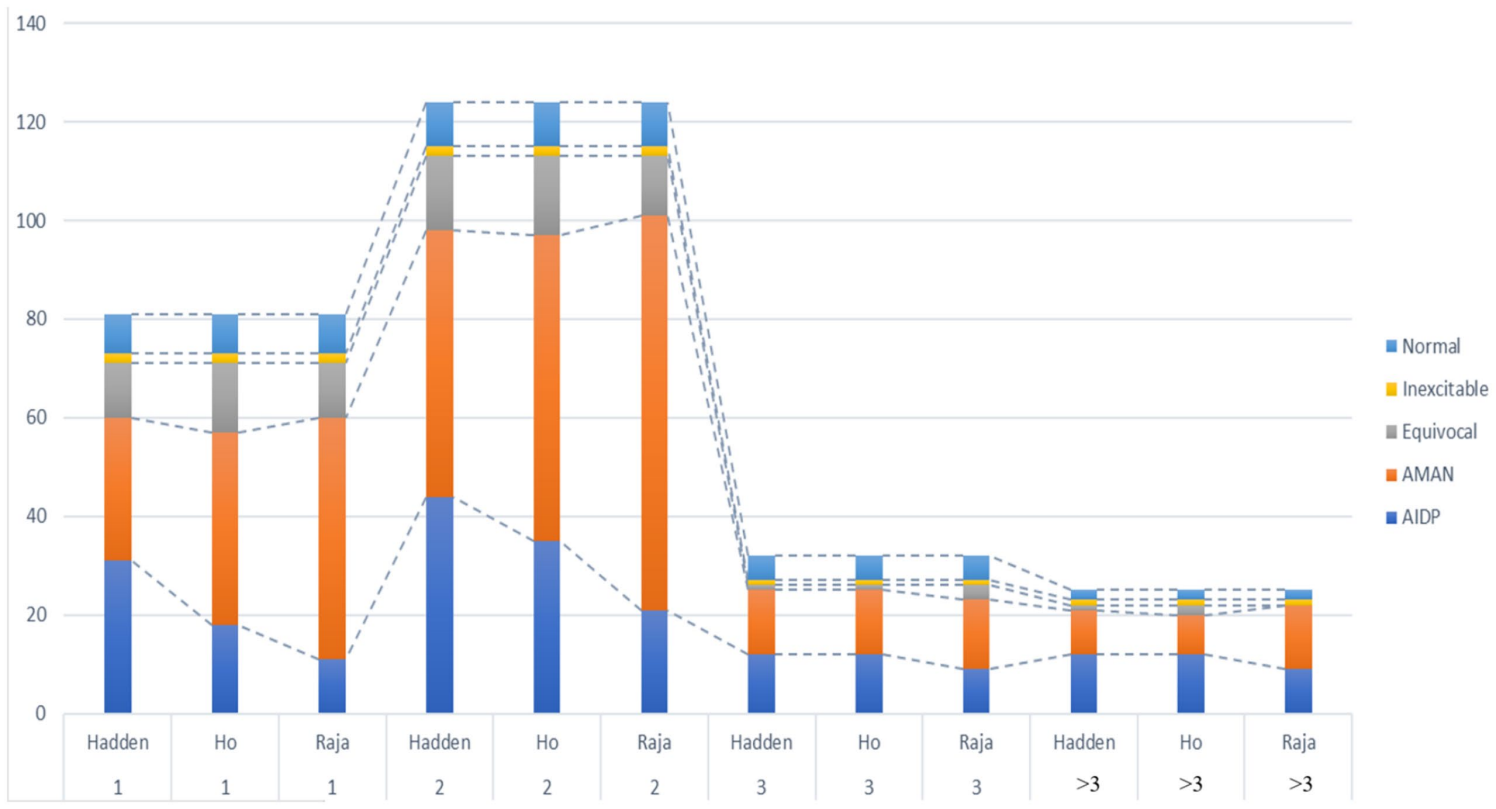
Comparison of different electrodiagnostic criteria at different post-onset time points (1 week, 2 weeks, 3 weeks, >3 weeks). Raja, Rajabally’s criteria; Hadden, Hadden’s criteria; Ho, Ho’s criteria; The *X*-axis indicates NCS examination timing, while the *Y*-axis shows the number of GBS patients per subtype.

Among the 262 patients, NCS examinations were performed at different time points post onset: 81 patients were tested in the 1st week, 124 in the 2nd week, 32 in the 3rd week, and the remaining 25 patients underwent the examination at time points beyond the 3rd week. Most patients (78.3%) completed the examination within the first 2 weeks after onset. Three electrophysiological criteria were applied to GBS patients who completed NCS. Within the first 3 weeks after onset, regardless of the classification criteria applied, the results consistently demonstrated a predominance of the AMAN subtype (see Figure 3). There were significant differences in the classification results (*p* = 0.003, *p* = 0.007) in the 1st and 2nd week. Hadden criteria tended to classify GBS patients as AIDP, while Rajabally criteria tended to classify GBS patients as AMAN. For GBS patients who underwent NCS beyond 3 weeks after onset, the electrophysiological subtype results obtained using the three classification criteria were relatively consistent (*p* = 0.718, *p* = 0.32) (see Supplementary Table 2 and Figure 4).

### 3.5 The correlation between sural sparing and different electrophysiological subtypes

Based on the typing results of these three electrophysiological classification criteria, it was consistently found that the proportion of sural sparing was significantly higher in AIDP patients than in AMAN patients (32.4–50% vs. 0.9–4%) (*p* < 0.001) (see Table 2); sural sparing was mainly observed in AIDP.

**Table 2:** Sural sparing pattern with different GBS subtypes.

Electrophysiological criteria	Subtype	Sural sparing		*χ* ^2^	*p-value*
		No	Yes		
Rajabally’s criteria	AIDP	25	25	63.464	<0.001
	AMAN	150	6		
Hadden’s criteria	AIDP	67	32	57.714	<0.001
	AMAN	104	1		
Ho’s criteria	AIDP	45	32	77.137	<0.001
	AMAN	121	1		

### 3.6 Correlation between conduction block and prognosis

According to the Rajabally criteria, among AMAN patients, 50 had conduction block and 106 did not. Among AIDP patients, 21 had conduction block and 29 did not (see Table 3). There was no difference in HFGS scores at admission and peak time between patients with and without conduction block within the AMAN and AIDP subtypes (all *p* > 0.05). However, compared with AMAN patients without conduction block, those with conduction block had lower HFGS scores at discharge (*p* = 0.012). No differences were observed in HFGS scores at admission, peak time, and discharge between AIDP patients with conduction block and AMAN patients with conduction block (all *p* > 0.05).

**Table 3:** Conduction block and clinical prognosis.

HFGS scores	AMAN with CB *N* = 50	AMAN without CB *N* = 106	AIDP with CB *N* = 21	AIDP without CB *N* = 29	*P-value*		
					AMAN with vs. without CB	AIDP with vs. without CB	AMAN vs. AIDP with CB
HFGS scores at admission	3.3 ± 0.74	3.34 ± 0.98	3.23 ± 0.88	3.07 ± 1.19	0.801	0.568	0.78
HFGS scores at peak	3.36 ± 0.83	3.58 ± 0.85	3.57 ± 0.81	3.38 ± 1.34	0.122	0.564	0.325
HFGS scores at discharge	2.64 ± 1.08	3.1 ± 1.05	2.85 ± 0.79	2.69 ± 1.31	***0.012***	0.606	0.352

CB, conduction block; HFGS, Hughes Functional Grading Scale.

The bold values indicate *p* < 0.05.

### 3.7 Changes in electrophysiological subtypes of GBS patients during follow-up

A total of 31 GBS patients underwent ≥ 2 NCS examinations: the first was performed within 2 weeks after onset, and the second between 3 and 8 weeks after onset. We used Hadden criteria to classify the initial NCS results of 31 GBS patients into AIDP (14 cases, 45.2%), AMAN (10 cases, 32.3%), and unclassified (7 cases, 22.5%). During the second NCS, a total of 11 cases exhibited subtype changes: three AIDP patients had resolution of CB recovery of prolonged terminal latency and normal recovery of CMAP amplitude, transitioning to a pattern of rapid recovery to normal electrophysiological status. Two AIDP patients had resolution of CB with CMAP amplitude decreasing to <80% of the lower normal limit and converted to AMAN subtype. Among the seven patients initially diagnosed as unclassified, five patients converted to AMAN subtype and one patient converted to AIDP subtype. The results of the second electrophysiological examination showed 11 cases classified as AMAN, 10 as AIDP, 1 as unclassified or unclassifiable type, and 3 with rapid recovery to normal electrophysiological status, the predominant new subtype was AMAN (see Figure 3).

## 4 Discussion

This study was the first to quantitatively compare three electrophysiological diagnostic criteria in a single cohort of GBS patients from China, which showed high inter-criterion consistency. AMAN was the predominant GBS subtype in our cohort regardless of the criterion used. The presence of CB lead to differences in GBS classification in the early stage (<3 weeks after symptom onset), whereas the early introduction of the sural sparing indicator and implementation of serial NCS (≥2 tests) enable more accurate subtype differentiation.

Currently, the electrophysiological criteria have certain limitations. The Ho criteria uses “exact time dispersion” to assess AIDP but lacks a clear definition of its degree. Most widely used Hadden criteria replaced “waveform dispersion” with “conduction block” to evaluate AIDP, but as AMAN patients may also have CB in early stage (6), some of these patients maybe mistakenly classified as AIDP or unclassified (23–25). Rajabally criteria (4) are stricter for AIDP diagnosis and permit reversible conduction block in AMAN patients. In this study, while the three electrophysiological classification criteria exhibited high consistency, their limitations were also confirmed-specifically reflected in significant differences in the proportions of each subtype, which is supported by Uncini et al.’s (15) findings. Rajabally’s criteria are recommended to improve AMAN diagnostic sensitivity, while Hadden’s criteria can be used as an alternative for balanced sensitivity and specificity. Based on the above findings, the differences in the electrophysiological subtype composition ratios among GBS patients in different regions (26–30) may undergo changes with variations in the adopted electrophysiological classification criteria, and may even lead to the reversal of primary and secondary subtypes. Therefore, it is necessary to conduct further verification using different classification criteria based on the electrophysiological data of GBS patients in different regions.

Regarding the limitations of electrophysiological classification criteria, some scholars hold that serial NCS enables more accurate electrophysiological classification (12, 14). Our research findings also confirm this theory. In our study, 31 patients underwent ≥2 serial NCS tests and were classified using Hadden criteria, 35% of these patients exhibited classification changes, which were attributed to two key factors: ① Disappearance of CB in some initially diagnosed AIDP patients—these patients either rapidly recovered to normal NCS or had further amplitude reduction, thus transforming into AMAN; ② Initially unclassified patients who met AMAN criteria during follow-up. This finding was consistent with the study of Yuki et al. (13). In this follow-up phase of this study, some patients had sustained decrease or absence of distal CMAP amplitude, while others showed rapid normalization (no increased time dispersion, prolonged latency in distal CMAP amplitude/distal motor latency, or delayed CB recovery). These findings, inconsistent with AIDP’s demyelination/remyelination, indicate AMAN is characterized by both axonal degeneration and reversible CB (possibly induced by anti-ganglioside antibodies targeting the axonal membrane at nodes of Ranvier). Notably, transient reversible CB without waveform dispersion can occur in GBS axonal subtypes, suggesting early electrophysiological subtyping errors (31). In current clinical practice, GBS patients often undergo NCS within 2 weeks after the onset of symptoms (32). This study found that there were significant differences in the classification results when different criteria were used to classify patients within 2 weeks, the Hadden criteria tended to classify GBS patients as AIDP, while the Rajabally criteria tended to classify GBS patients as AMAN. CB usually disappears after 3 weeks, so the results of these three classification criteria were more consistent after 3 weeks. Therefore, the electrophysiological classification of GBS needs to take both the classification criteria and the timing of NCS examination into account and further improve the accuracy of classification through a series of electrophysiological examinations (14, 15). Given our study findings—specifically, that CB typically resolves by 3 weeks after symptom onset, and that the classification results of the three criteria become more consistent at this time point—it can be hypothesized that performing NCS at ≥3 weeks post-symptom onset may yield more consistent GBS subtype classification results. For patients with early disease onset (<3 weeks post-symptom onset), serial NCS (i.e., ≥2 examinations) is further recommended to minimize the risk of misclassification. Nevertheless, this hypothesis has not been fully validated. The current optimal NCS time window remains unclear and still requires further confirmation and refinement of the specific time range through large-sample, prospective studies.

Sural sparing also aids in the diagnosis and electrophysiological classification of GBS (18). Sural sparing as a characteristic manifestation of sensory nerve conduction in patients with GBS was reported to be suggestive of AIDP (33), and may also occur in AMAN (11, 34, 35). Our study observed that both AIDP and AMAN present with the sural sparing pattern, with the highest proportion in AIDP patients. Sural sparing may provide a reference for the subtype classification of GBS, but it is significantly affected by the timing of examinations, and its application value should be comprehensively evaluated in combination with disease duration, which is consistent with previous studies (11, 34–37). Sural sparing can be used as a supplementary indicator to distinguish AIDP from AMAN. However, the overall incidence of sural sparing in our study (13.7%) was much lower than that reported in other studies [50% (37), 30% (38)], possibly due to regional differences in GBS subtype distribution (39) –as our study population was from an AMAN-predominant region, whereas AIDP is predominant in European and American countries.

Electrophysiological classification of GBS is important for predicting its clinical course and determining treatment in patients with GBS. In our study, AMAN patients with CB achieved a relatively favorable therapeutic response at discharge, which may suggest a more promising short-term prognosis and necessitates regular follow-up after hospital discharge. However, given the retrospective design, electrophysiological examinations performed across a broad disease window and short-term follow-up, this finding should be considered exploratory. This finding was consistent with Niu et al.’s (40) research. This may relate to distinct pathophysiological mechanisms of CB in AIDP and AMAN: In AIDP, severe demyelination blocks the transmission of some nerve fibers and impulses, causing CB (41); in AMAN, autoimmune antibodies GM1/GD1a bind to Ranvier segment, forming nerve myelin sheath attack complexes. This leads to the loss of voltage-gated sodium channels and detachment of adjacent myelin sheath ring (42), resulting in reversible motor nerve CB. Since demyelination repair requires much longer functional recovery time than sodium channel reactivation, the above mechanisms support our results. However, other studies (43, 44) did not find an association between CB and prognosis in GBS. This may be related to factors such as the study population and classification criteria, which also underscores the need to develop region-specific prognostic indicators.

Due to the retrospective nature of this study, there were some limitations. ① Wide variability in the timing of nerve conduction studies relative to symptom onset and electrophysiological examinations were performed across a broad disease window. ②Single-center retrospective design with a small number of patients undergoing serial NCS, limiting generalizability; ③ Lack of a gold standard for GBS electrodiagnosis; ④Short-term prognosis assessment (discharge HFGS) without 6-month+ follow-up, precluding analysis of long-term outcomes.

## 5 Conclusion

AMAN is the main GBS subtype in our cohort from northern China. Different electrophysiological criteria and NCS timing affect GBS subtyping, with more consistent results at ≥3 weeks post-onset. Sural sparing and serial NCS enhance subtype classification accuracy. Limitations include the single-center retrospective design and short-term follow-up, so conclusions need validation in large-scale prospective studies.

## Data Availability

The original contributions presented in the study are included in the article/Supplementary material, further inquiries can be directed to the corresponding author.
