# Supplementary material for: Re-evaluation of different electrophysiological criteria for Guillain-Barré syndrome in a single cohort from China
Source: Front Neurol. 2026 Mar 11;17:1766901. doi: 10.3389/fneur.2026.1766901 (PMC13013526; doi:10.3389/fneur.2026.1766901)
Supplement: Supplementary file 1 [file Table_1.docx]

Supplementary Table 1 Three different electrophysiological criteria for GBS

| Parameters | **Ho** | **Hadden** | **Rajabally** |
| --- | --- | --- | --- |
| AIDP |  |  |  |
| MCV | <90% LLN  <85% if D-amp <50% | <90%LLN  <85% if Damp <50% | <70% LLN |
| DL | >110% ULN  >120% if D amp <LLN | >110% ULN  >120% if d-amp <LLN | >150%ULN |
| TD | Unequivocal | Not considered | Not considered |
| CB | Not considered | P-D amp ratio < 0.5 D amp > 20% LLN | <0.7 P-D amp ratio in two nerves with an additional parameter |
| F wave | >120%ULN | >120%ULN | >120% ULN  >150% ULN if distal CMAP <50% LLN  Absent in two nerves with dCMAP >20% LLN with an additional parameter |
| AMAN | No AIDP  D amp <80%LLN | No AIDP  D amp <80%LLN | No AIDP  D amp <80%LLN P-D amp ratio < 0.7 |
| Inexcitable | Not considered | Absent DCMAP in all nerves  Present in only one nerve with<10%LLN | Absent D CMAP in all nerves  Present in only one nerve with <10%LLN |
| Equivocal | Not considered | Not fitting criteria | Not fitting criteria |

AIDP Acute Inflammatory Demyelinating Polyneuropathy. MCV Mean Conduction Velocity, DL Distal Latency. TD Temporal Dispersion, CB Conduction Block, SNAP Sensory Nerve Action Potential, LLN Lower Limit Normal, ULN Upper Limit Normal

Table 2 Comparison of different electrodiagnostic criteria at different time

| time（weeks） | criteria | AIDP | AMAN | Equivocal | Inexcitable | Normal | χ^2^ | *P*-value |
| --- | --- | --- | --- | --- | --- | --- | --- | --- |
| 1 | Hadden | 31 | 29 | 11 | 2 | 8 | 15.928 | **0.003** |
| 1 | Ho | 18 | 39 | 14 | 2 | 8 |  |  |
| 1 | Rajabally | 11 | 49 | 11 | 2 | 8 |  |  |
| 2 | Hadden | 44 | 54 | 15 | 2 | 9 | 14.093 | **0.007** |
| 2 | Ho | 35 | 62 | 16 | 2 | 9 |  |  |
| 2 | Rajabally | 21 | 80 | 12 | 2 | 9 |  |  |
| 3 | Hadden | 12 | 13 | 1 | 1 | 5 | 2.097 | 0.718 |
| 3 | Ho | 12 | 13 | 1 | 1 | 5 |  |  |
| 3 | Rajabally | 9 | 14 | 3 | 1 | 5 |  |  |
| >3 | Hadden | 12 | 9 | 1 | 1 | 2 | 4.692 | 0.320 |
| >3 | Ho | 12 | 8 | 2 | 1 | 2 |  |  |
| >3 | Rajabally | 9 | 13 | 0 | 1 | 2 |  |  |
